# Psychometric properties of an Arabic translation of the Big Three Perfectionism Scale–Short Form (BTPS-SF) in a community sample of adults

**DOI:** 10.1186/s12888-023-05427-y

**Published:** 2023-12-11

**Authors:** Feten Fekih-Romdhane, Radoslaw Rogoza, Rabih Hallit, Diana Malaeb, Fouad Sakr, Mariam Dabbous, Toni Sawma, Sahar Obeid, Souheil Hallit

**Affiliations:** 1 The Tunisian Center of Early Intervention in Psychosis, Department of Psychiatry “Ibn Omrane”, Razi hospital, Manouba, 2010 Tunisia; 2 Faculty of Medicine of Tunis, Tunis El Manar University, Tunis, Tunisia; 3 University of Economics and Human Sciences in Warsaw, Warsaw, Poland; 4 Department of Psychology, University of Lleida, Lleida, Spain; 5 School of Medicine and Medical Sciences, Holy Spirit University of Kaslik, P.O. Box 446, Jounieh, Lebanon; 6 Department of Infectious Disease, Bellevue Medical Center, Mansourieh, Lebanon; 7 Department of Infectious Disease, Notre Dame des Secours University Hospital, Postal code 3, Byblos, Lebanon; 8 College of Pharmacy, Gulf Medical University, Ajman, United Arab Emirates; 9 School of Pharmacy, Lebanese International University, Beirut, Lebanon; 10 School of Arts and Sciences, Social and Education Sciences Department, Lebanese American University, Jbeil, Lebanon; 11 Psychology Department, College of Humanities, Effat University, Jeddah, 21478 Saudi Arabia; 12 Applied Science Research Center, Applied Science Private University, Amman, Jordan

**Keywords:** Perfectionism, Self-critical perfectionism, Rigid perfectionism, Narcissistic perfectionism, Psychometric properties, Arabic

## Abstract

**Background:**

Despite the high clinical relevance of the perfectionism construct as a transdiagnostic contributor to different mental health symptoms, and the recent burgeoning of research in this area across cultures in the past two decades, the Arab region was one of the cultural settings experiencing the slowest progress in this line of research. This study aimed to make a meaningful contribution to the literature by validating an Arabic-language version of the 16-item Big Three Perfectionism Scale–Short Form (BTPS-SF). In particular, we sought to examine structure and concurrent validity, internal consistency, and measurement invariance across gender groups.

**Method:**

A web-based, convenience sampling method was adopted to collect a sample of Arabic-speaking adults from the general population of Lebanon (N = 515; aged 27.55 ± 10.92 years; 69.9% females). The forward-backward method was applied in translating the Arabic version of the BTPS-SF.

**Results:**

The examination of the internal structure, using Confirmatory Factor Analysis (CFA), demonstrated that the three-factor model (i.e., rigid, self-critical, and narcissistic perfectionism) fitted well to the data. All three factors of the Arabic 16-item BTPS-SF yielded excellent reliability estimates, with both Cronbach’s alpha and McDonald’s omega ranging from 0.83 to 0.86. Multi-group CFA revealed that fit indices showed no significant difference in model fit at the configural, metric, and scalar levels, thus suggesting that the factor loadings, pattern structure, and item intercepts are invariant across gender groups. Finally, BTPS-SF subscales scores correlated positively with psychological distress (i.e., depression, stress and anxiety), and inversely with subjective well-being, indicating an acceptable concurrent validity.

**Conclusion:**

The present findings allow us to conclude that the Arabic BTPS-SF permits to capture reliably and validly three main factors of perfectionism. We hope that providing this psychometrically sound scale will encourage its large use not only in empirical research, but also in clinical applications, including psychological screening and treatment monitoring.

## Introduction

The concept of perfectionism refers to a multidimensional personality tendency to set and endeavour to attain unrealistic, overly high personal standards, strive for flawlessness, and critically evaluate the self and others [1, 2]. Cross-temporal meta-analysis findings showed that levels of perfectionism are linearly on the rise [3], which is mainly due to rapid societal changes (e.g., impact of social media on self- and socially-oriented perfectionism) and the early childhood adoption of perfectionistic thought and behaviour patterns [4–9]. Although perfectionism may take both adaptive and maladaptive forms, it has gained growing attention in psychological research and clinical applications in recent years because of its negative and far-reaching effects on mental health, behavioural, social, and life outcomes [10]. Indeed, perfectionism has consistently proven to play a key role in a wide range of psychopathology (for meta-analysis, see [11]). For instance, there is strong evidence that perfectionistic concerns represent significant risk factors for anxiety [12] and depressive [13] symptoms, perinatal depression [14], stress [15, 16], burnout [17], disordered eating [18–20], obsessive-compulsive disorder [21], different maladaptive personality traits, particularly narcissism [22], body dysmorphic symptoms [23], sleep problems [24], and even suicide ideation and attempts [9]. Additional findings suggested that perfectionism has detrimental effects on daily functioning [25] and subjective well-being [26–29].

In light of its association with many psychopathologies as either a precursor or a maintaining factor, perfectionism has been considered in recent years to be a transdiagnostic factor [30, 31]. A potential asset of a transdiagnostic approach is that delivering psychological interventions in which the focus is on transdiagnostic mechanisms may have effects on the different forms of psychopathology it is related to [32]. In this regard, a number of empirical studies have proven the effectiveness of psychological interventions aimed at remediating perfectionism in both reducing perfectionism and addressing other mental problems (e.g., symptoms of depression, anxiety and eating disorders) [33]. All these observations emphasize the crucial importance of adequate psychometric assessment of the multidimensional perfectionism construct according to latest advances [34].

Over the past three decades, different models and their corresponding measures of perfectionism have been proposed [34]. The self-report Frost’s Multidimensional Perfectionism Scale [1] is composed of five dimensions (i.e., concern over mistakes, parental expectations, parental criticism, personal standards, and doubts about actions). Another commonly used measure, the Hewitt and Flett’s Multidimensional Perfectionism Scale [2], incorporates three dimensions of perfectionism (i.e., self-oriented perfectionism, other-oriented perfectionism, and socially prescribed perfectionism), that were combined later into two forms labeled perfectionistic strivings and perfectionistic concerns [35]. These measures have been criticized for their limited utility, the overlapping content of some items, the inconsistent factor-item distributions and factor numbers, as well as the presence of second-order manifestations in some perfectionism dimensions (e.g., those on parenting) [36, 37].

More recently, Smith et al. [38] developed a comprehensive self-report measure, i.e. the Big Three Perfectionism Scale (BTPS-45), with three higher-order global factors (rigid perfectionism, self-critical perfectionism, and narcissistic perfectionism) and 10 lower-order facets (self-oriented perfectionism, concern over mistakes, self-worth contingencies, self-criticism, doubts about actions, hypercriticism, other-oriented perfectionism, socially prescribed perfectionism, grandiosity, entitlement). Rigid perfectionism refers to requiring “flawless performance from the self” and insisting on everything being without faults or errors [38]. Self-critical perfectionism reflects negative responses to and concerns about flawed or imperfect performance and the belief that others demand one to be perfect [39]. Narcissistic perfectionism can be defined as a tendency to demand perfection from others (other-oriented) in an entitled, hypercritical, and grandiose way [38]. In a later revision in 2020, the BTPS-45 was shortened to 16 scored items (the BTPS-Short Form; BTPS-SF) [40], while maintaining the three higher order factors proposed in the original full-length form. In addition, the validity and reliability of this short form were confirmed in a sample of university students [40]. Even though having been developed only recently, the BTPS-16 has gained considerable research interest, and was adapted to other languages and contexts, including Italian [41, 42] and Turkish [43]. The BTPS-45 has been validated in Arabic in 2023 by a Jordanian team [44]. However, as far as we are aware of, no empirical study has examined the reliability and validity of the BTPS-SF in the Arabic language and context.

### Rationale of the present study

Despite the high clinical relevance of the perfectionism construct as a transdiagnostic contributor to different mental health symptoms, and the recent burgeoning of research in this area across cultures in the past two decades, the Arab region was one of the cultural settings experiencing the slowest progress in this line of research. We are aware of only a very few publications on this topic in the Arab region and culture (e.g., Saudi Arabian students studying in United States Universities [45], Egyptian adolescents [46], Jordanian students [44], Lebanese adults [47]). This might partly be due to the lack of convenient to use and psychometrically valid measures able to capture perfectionism in the Arab context. Although an Arabic version of the BTPS-45 was made available, some studies have applied an Arabic translation of the BTPS-SF without verifying its psychometric properties (e.g., [47–50]). The short-form version is economical, which represents a significant advantage particularly in the Arab low-resource research settings. In addition, because it is time-efficient, the BTPS-SF is valuable when respondents have to complete large-scale and/or multiple time-point surveys, which is rather a common occurrence in research involving transdiagnostic factors. Making available a reliable and valid instrument for measuring perfectionism could open future research to assess this transdiagnostic construct in clinical and nonclinical settings, and may offer novel perspectives for prevention and early intervention strategies in different mental health conditions. In addition, there is a need for a cost-effective and cross-culturally valid measure that can enable researchers to investigate and compare the level of perfectionism between different societies, and can allow to perform multi-country longitudinal research on perfectionism that help advance our understanding of the construct. For all these considerations, this study aimed to make a meaningful contribution to the literature by validating an Arabic-language version of the BTPS-SF in a sample of non-clinical adults from Lebanon. In particular, we sought to examine structure and concurrent validity, internal consistency, and measurement invariance across gender groups. We hypothesized that the Arabic BTPS-SF will confirm the originally proposed three-factor structure model, be invariant across gender, and demonstrate a good composite reliability. We also expected that the Arabic version will show good concurrent validity through an examination of its correlations with psychological distress and well-being measures.

## Methods

### Procedures

All data were collected via a Google Forms link; the sample was recruited conveniently between February and March 2023. The project was advertised on social media and included an estimated duration. Inclusion criteria for participation included: (1) being of a resident and citizen of Lebanon, (2) aged 18 years and above, (3) having access to the Internet, and (4) willing to participate in the study. Excluded were those who refused to fill out the questionnaire. Excluded were those who refused to fill the survey. After providing digital informed consent, participants were asked to complete the instruments described above, which were presented in a pre-randomised order to control for order effects. The survey was anonymous and participants completed the survey voluntarily and without remuneration [51].

### Measures

#### Big Three Perfectionism Scale - Short Form (BTPS-SF)

This scale is composed of 16 items, scored on a 5-point Likert scale, “Strongly disagree to Strongly agree” [52]. It yields three subscales: rigid perfectionism (i.e. “I have a strong need to be perfect”), self-critical perfectionism (i.e. “The idea of making a mistake frightens me”) and narcissistic perfectionism (i.e. “I get frustrated when other people make mistakes”). Higher scores reflect higher perfectionism in the three aspects. We used the Arabic version of the scale, which was previously used [53].

#### Depression Anxiety and Stress Scale- 8 items (DASS-8)

Validated in Arabic [54], this scale is composed of eight items (i.e. “I was worried about the situations in which I might panic and make a fool of myself” that measure depression (3 items), anxiety (3 items) and stress (2 items). Questions are rated on a 4-point Likert scale (“0 = does not apply to me to “3 = always applies to me”). Higher scores reflect more psychological distress (ω = .89 in this study).

#### WHO-wellbeing scale

Validated in Arabic [55], this scale is composed of 5 items (i.e. “in the last two weeks, I have felt cheerful in good spirits”) scored on a 6-point Likert scale (“0 = at none time to 5 = all of time”), with higher scores reflecting better wellbeing [56] (ω = 0.93 in this study).

#### Demographics

Participants were asked to provide their demographic details consisting of age, sex, marital status, education level and household crowding index (defined as number of persons / number of rooms in the house excluding the kitchen and bathrooms [57]).

### Analytic strategy

#### Confirmatory factor analysis (CFA)

There were no missing responses in the dataset. We used data from the total sample to conduct a CFA using the Mplus v.8.2 software. As a rule of thumb, simulation studies show that with normally distributed indicator variables and no missing data, a reasonable sample size for a simple confirmatory factor analysis model is about N = 150 [58], which was exceeded in our sample. Our intention was to test the original model of the BTPS-SF scale (i.e., three-factor model). Parameter estimates were obtained using the maximum likelihood. To evaluate model fit, we relied on the root mean square error of approximation (RMSEA) and the comparative fit index (CFI). Values < 0.08 for RMSEA, and > 0.90 for CFI indicate good fit of the model to the data [59].

#### Gender invariance

To examine gender invariance of BTPS-SF scores, we conducted a multi-group CFA [60] using the total sample on the measurement final model. Configural invariance implies that the latent scales variable(s) and the pattern of loadings of the latent variable(s) on indicators are similar across gender (i.e., the unconstrained latent model should fit the data well in both groups). Metric invariance implies that the magnitude of the loadings is similar across gender; this is tested by comparing two nested models consisting of a baseline model and an invariance model. Lastly, scalar invariance implies that both the item loadings and item intercepts are similar across gender and is examined using the same nested-model comparison strategy as with metric invariance [61]. For configural invariance, we considered same criteria as described above and for metric and scalar invariance, we deemed ΔCFI ≤ 0.010 and ΔRMSEA ≤ 0.015 as evidence of invariance [60].

#### Further analyses

Composite reliability in both subsamples was assessed using McDonald’s ω and Cronbach’s, with values greater than 0.70 reflecting adequate composite reliability [62]. The three perfectionism scores were considered normally distributed since the skewness and kurtosis values varied between ± 1 [63] as follows: rigid perfectionism (S = − 0.206; K = − 0.439), self-critical perfectionism (S = − 0.190; K = − 0.389) and narcissistic perfectionism (S = 0.140; K = − 0.427). Therefore, to assess concurrent validity, we examined bivariate correlations between the three scores and the other scales using the Pearson test. Based on Cohen (1992) [64], values ≤ 0.10 were considered weak, ~ 0.30 were considered moderate, and ~ 0.50 were considered strong correlations.

## Results

### Participants

Five hundred fifteen participants participated in this study, with a mean age of 27.55 ± 10.92 years and 69.9% females. Furthermore, 26.8% were married and 83.7% had a university level of education. The mean household crowding index was 1.15 ± 0.57.

### Confirmatory factor analysis of the Big Three Perfectionism scale

CFA indicated that fit of the three-factor model of the BTPS-SF scale were modest: χ^2^ = 487.71, df = 101 (*p* < .001), RMSEA = 0.065 (90% CI 0.049, 0.081), SRMR = 0.072, CFI = 0.860, TLI = 0.834. When adding correlations between items 2–3, 5–6 and 12–13, the fit indices improved as follows: χ^2^ = 373.03, df = 98 (*p* < .001), RMSEA = 0.074 (90% CI 0.066, 0.082), SRMR = 0.064, CFI = 0.901, TLI = 0.878. The standardised estimates of factor loadings were all adequate (Table 1).

**Table 1:** Standardized loading factors of the Big Three Perfectionism Scale-Short Form (BTPS-SF) items on the total sample

	Loading factor
Factor 1: Rigid perfectionism	
1. I have a strong need to be perfect	0.72
2. It is important to me to be perfect in everything I attempt	0.70
3. Striving to be as perfect as possible makes me feel worthwhile	0.70
4. My opinion of myself is tied to being perfect	0.85
Factor 2: Self-critical perfectionism	
5. The idea of making a mistake frightens me	0.68
6. When I notice that I have made a mistake, I feel ashamed	0.65
7. I have doubts about everything I do	0.65
8. I judge myself harshly when I don’t do something perfectly	0.75
9. I feel disappointed with myself, when I don’t do something perfectly	0.79
10. People are disappointed in me whenever I don’t do something perfectly	0.70
Factor 3: Narcissistic perfectionism	
11. I expect those close to me to be perfect	0.68
12. I am highly critical of other people’s imperfections	0.61
13. I feel dissatisfied with other people, even when I know they are trying their best	0.64
14. It bothers me when people don’t notice how perfect I am	0.72
15. I deserve to always have things go my way	0.66
16. I know that I am perfect	0.60

### Measurement invariance

As reported in Table 2, although the estimates of the CFI were slightly below accepted threshold, the comparison across models suggested that configural, metric, and scalar invariance was supported across gender. The results showed that there was no statistically significant difference in latent mean scores between males and females in neither BTPS-SF dimensions (Table 3).

**Table 2:** Measurement Invariance of the Big Three Perfectionism Scale-Short Form (BTPS-SF) across gender in the total sample

Model	χ²	df	CFI	RMSEA	Model Comparison	Δχ²	ΔCFI	ΔRMSEA	Δdf	p
Configural	477.14	196	0.899	0.075						
Metric	495.70	209	0.897	0.073	Configural vs. metric	18.56	0.002	0.002	13	0.137
Scalar	516.61	222	0.894	0.072	Metric vs. scalar	20.91	0.003	0.001	13	0.074

*Note*: CFI = Comparative fit index; RMSEA = Steiger-Lind root mean square error of approximation; SRMR = Standardised root mean square residual

### Composite reliability

Composite reliability of scores was adequate in the total sample for the rigid perfectionism (ω = 0.85 / α = 0.85), self-critical perfectionism (ω = 0.86 / α = 0.86), and narcissistic perfectionism (ω = 0.83 / α = 0.83) subscales.

**Table 3:** Comparison between genders in terms of the Big Three Perfectionism Scale-Short Form (BTPS-SF) subscales’ scores in the total sample

	Rigid perfectionism	Self-critical perfectionism	Narcissistic perfectionism
Gender			
Males	12.24 ± 3.50	16.79 ± 4.69	15.76 ± 4.36
Females	12.32 ± 3.46	17.43 ± 4.90	15.32 ± 4.55
*t*	0.251	1.373	1.024
*df*	513	513	513
*p*	0.802	0.170	0.306

### Concurrent validity

Higher rigid and self-critical perfectionism subscores were significantly correlated with lower well-being. All three perfectionism subscores were significantly associated with higher psychological distress (Table 4).

**Table 4:** Correlations of the Big Three Perfectionism Scale-Short Form (BTPS-SF) subscores with the other measures in the total sample

	Mean ± SD	1	2	3	4	5	6
1. Rigid perfectionism	12.30 ± 3.47	1					
2. Self-critical perfectionism	17.23 ± 4.84	0.60***	1				
3. Narcissistic perfectionism	15.45 ± 4.49	0.55***	0.58***	1			
4. Wellbeing	14.08 ± 5.60	− 0.13**	− 0.23***	− 0.07	1		
5. Psychological distress	11.16 ± 6.72	0.09*	0.33***	0.25***	− 0.38***	0.02	1

*p < .05; **p < .01; ***p < .001; values reflect Pearson correlation coefficients

## Discussion

In the current study, we sought to validate the Arabic version of the 16-item BTPS-SF in a sample of native Arabic-speaking adults from the general population of Lebanon. Our findings revealed that the factorial validity of the Arabic scale was supported, with all 16 items being grouped under three factors. A very good internal consistency of the Arabic BTPS-SF was demonstrated through high values of Cronbach’s alpha and McDonald’s omega, and measurement invariance was supported across gender. Evidence for concurrent validity was obtained through correlations of the BTPS-SF subscales scores in the expected directions with psychological distress and well-being scores.

The examination of the internal structure, using confirmatory factor analysis, demonstrated that the three-factor model fitted well to the data. Our results provide additional evidence to support the three-factor representation of perfectionism originally proposed in both the long [38] and short [40] forms of the BTPS (i.e., rigid, self-critical, and narcissistic perfectionism). The three-factor structure of the BTPS-SF was also obtained and adopted in other linguistic versions, such as the Turkish [43] and Italian [41]. These findings further reinforce the conceptualization of perfectionism as a multidimensional construct (e.g., [1, 2, 65]). Furthermore, all three factors of the Arabic 16-item BTPS-SF yielded excellent reliability estimates, with both Cronbach’s alpha and McDonald’s omega ranging from 0.83 to 0.86 in our sample of community adults. In the original validation study, Cronbach’s alpha coefficient values for the three BTPS-SF perfectionism factors were high, ranging from 0.78 to 0.86 in two samples of Canadian university students [40]. Similar high levels of internal consistency for all of the big three perfectionism dimensions were also reported in other populations and countries (e.g., alpha coefficients of 0.75-0.86 in Turkish community adults [43], 0.83-0.89 in Italian university students [41]).

Beyond factorial validity and internal consistency, multi-group CFA revealed that fit indices showed no significant difference in model fit at the configural, metric, and scalar levels, thus suggesting that the factor loadings, pattern structure, and item intercepts are invariant across gender groups. Although it has been recommended that psychometric properties including measurement invariance of the BTPS should be investigated for different nationalities for a better generalizability [38], most of the previous validation studies did not address this point (e.g., [41, 43, 66]). As such, only scant evidence exists so far to support measurement invariance of the BTPS for gender groups. A few studies were able to demonstrate measurement invariance of the long form of the BTPS between males and females in various populations (e.g., multi-ethnic undergraduates [38], Turkish undergraduates [67]). However, we could find no studies that have established this psychometric property for the short form of the scale.

Finally, BTPS-SF subscales scores correlated positively with psychological distress (i.e., depression, stress and anxiety), and inversely with subjective well-being, indicating an acceptable concurrent validity of the Arabic version. In particular, self-critical perfectionism showed the highest relationship with these outcomes. These results are in line with the original validation study which revealed similar correlational patterns of the BTPS-SF with depression, anxiety, stress, and other maladaptive outcomes, thereby attesting to the criterion validity of the scale [40]. Likewise, the psychometric study of the Turkish BTPS-SF found that perfectionism dimensions were relatively correlated with a range of psychopathology, including depression, anxiety, obsessive compulsive disorder and maladaptive personality traits [43]. Consistently, perfectionism was show to predict negative psychological indicators, such as increased depression, anxiety, and stress symptoms, and less satisfaction with life across cultures [68]. Strong meta-analytic evidence drawn from longitudinal studies [12, 69] and meta-synthesis of qualitative studies [70] concluded that the multidimensional perfectionism serves as a risk factor for depressive and anxiety symptoms. As for findings on self-critical perfectionism, they concur with those of previous studies [48, 67, 71], suggesting this component as a critical factor in the prediction of psychopathology.

### Limitations and research perspectives

Our study is not without limitations. We referred to a web-based design, which have mostly attracted young and female respondents. This might limit the generalizability of our conclusions. In addition, our study used an Arabic-speaking adult sample from a single Middle Eastern Arab country (i.e. Lebanon). Additional cross-national research should explore the reliability and validity of the Arabic BTPS-SF and confirm its robustness in other countries and cultural settings (e.g., North Africa). One important psychometric quality of the BTPS-SF, i.e. test–retest reliability, has not been investigated in the context of the present study and still needs to be explored in future research. Indeed, test–retest stability is salient for the perfectionism construct, as it is theoretically proposed as a trait that remains invariant across time [2]. Future studies are also still required to examine construct validity of the Arabic BTPS-SF, and how it relates to other perfectionism scales (such as the self-report Frost’s Multidimensional Perfectionism Scale [1]).

### Study implications

Overall, the present findings indicate that the Arabic version of the BTPS-SF is valid, reliable, and suitable for application among Arabic-speaking adult populations to detect perfectionism in Arab contexts. Multi-group analyses showed that the Arabic BTPS-SF could appropriately measure perfectionism in both males and females. This suggests that the Arabic BTPS-SF can be used in future studies to draw robust conclusions about latent mean comparisons between genders on the three-factor model of perfectionism. Correlation findings between perfectionism and study variables further confirm the previous assumptions that perfectionism dimensions are related to several psychopathological processes [11], and could be involved in their treatment [33], including in Arab settings. The adaptation and validation of the BTPS-SF in the Arabic language and culture is important and valuable in terms of its ability to shed light on perfectionism both as a primary presenting problem and as a factor accounting for the development and maintenance of different psychopathologies. Furthermore, offering a psychometrically sound perfectionism scale to Arab researchers will hopefully contribute to emerging local and cross-cultural research on perfectionism in Arab countries.

## Conclusion

The present findings allow us to conclude that the Arabic BTPS-SF permits to capture reliably and validly three main factors of perfectionism. Researchers and practitioners working in Arab settings can now benefit from this self-report measure that it is short, simple to use, quick to administer, and of low cost. We hope that providing this psychometrically sound scale will encourage its large use not only in empirical research, but also in clinical applications, including psychological screening and treatment monitoring. This would expand our knowledge of cross-cultural conceptions of perfectionism, and how each dimension relates to psychopathology in Arab populations.

## Data Availability

All data generated or analyzed during this study are not publicly available due the restrictions from the ethics committee, but are available upon a reasonable request from the corresponding author (SH).
